# Parthenolide: pioneering new frontiers in hematological malignancies

**DOI:** 10.3389/fphar.2025.1534686

**Published:** 2025-04-15

**Authors:** Elmira Zarei, Ayda Zarei, Azadeh Omidkhoda

**Affiliations:** ^1^ Department of Hematology and Blood Banking, School of Allied Medical Sciences, Tehran University of Medical Sciences, Tehran, Iran; ^2^ Department of Laboratory Sciences, School of Paramedical Sciences, Shiraz University of Medical Sciences, Shiraz, Iran

**Keywords:** hematological malignancy, parthenolide, herbal extract, leukemia, NF-kB

## Abstract

Hematological malignancies are the fifth most prevalent cancer category in developed countries, presenting significant treatment challenges. The side effects of chemotherapy and its non-selective nature have led researchers to explore combination therapies to enhance efficacy and improve patient outcomes. Parthenolide (PTL) emerges as a promising candidate, being the first small molecule identified for its selective action against leukemic stem cells. This article provides an overview of PTL’s effects and its potential as a complementary treatment for hematological malignancies. A systematic search in PubMed, Scopus, Embase and Web of Science identified relevant studies using keywords and MeSH terms. Peer-reviewed studies examining PTL’s mechanisms and therapeutic potential were included, while non-hematological studies and those lacking rigor were excluded. PTL targets key signaling pathways to combat malignancy, inhibiting NF-κB and signal transducer and STAT3, promoting ROS production, and activating p53. These mechanisms contribute to its effectiveness in treating hematological malignancies. Approaches incorporating PTL or its derivatives show promise in increasing leukemic cell sensitivity to existing therapies and reducing resistance likelihood. Additionally, studies indicate that other drugs can synergistically enhance PTL’s ability to eliminate leukemic cells. In conclusion, PTL represents a promising avenue for improving therapeutic outcomes in hematological malignancies, warranting further investigation into its mechanisms and potential in combination therapies.

## 1 Introduction

Blood malignancies encompass a variety of diseases that affect the blood, bone marrow, and associated organs. They contribute to approximately 1.2 million new cases globally, accounting for about 7% of all newly diagnosed cancers (Auberger et al., 2020). They can be classified as leukemia or lymphoma, each of which can be managed through a range of treatment options, such as chemotherapy, targeted therapies, immunotherapies and immune checkpoint inhibitors (Auberger et al., 2020). Chemotherapy, as the most used therapy, has a lot of complications such as; neuropathy (Jongen et al., 2015), pancytopenia (Van Tiel et al., 2005), susceptibility to infection (Rusu et al., 2018), fever, malaise and fatigue (Khalifa et al., 2021). Malignant hematopoietic cells often develop strategies to survive treatment, including acquiring mutations and modulating signaling pathways related to apoptosis, autophagy, metabolism, and epigenetic changes. These adaptations contribute to therapy-induced resistance (Auberger et al., 2020). Managing relapsed and refractory disease is highly challenging, with the inability to control the disease at this stage being the primary cause of mortality among patients with hematologic malignancies (Hofmann et al., 2023). Due to complications of chemotherapy and the rate of relapse, there has been growing interest in plant-derived natural compounds with potential anti-tumor properties, prompting efforts to identify their molecular targets (Xu et al., 2015). Herbal extracts have proven valuable for developing therapeutic agents for leukemia and lymphoma, offering a promising source for new drug candidates (Bahmani et al., 2022). Additionally, their synergistic effects with standard chemotherapeutic drugs underscore their significance in modern chemoprevention and chemotherapy research (Cheon, 2021; Pezzani et al., 2019; Lin et al., 2020).

Parthenolide is a sesquiterpene lactone extracted from the leaves of the medicinal herb Tanacetum parthenium. It possesses an α-methylene-γ-lactone ring and an epoxide group, enabling it to engage with the nucleophilic sites of various biological molecules (Juliana et al., 2010; Mannelli et al., 2015; Agatonovic-Kustrin and Morton, 2018). PTL has been a traditional herbal medicine for centuries, valued for its anti-inflammatory and anti-migraine properties (Kwok et al., 2001a; Materazzi et al., 2013). Lately, PTL has garnered attention for its anticancer properties, with various preclinical studies examining its effects on cancer cells from blood cancers and solid tumors (Diamanti et al., 2013; Spagnuolo et al., 2013; Siveen et al., 2017). PTL is remarkable for its ability to induce cell death in cancer cells while leaving normal cells unharmed (Czyz et al., 2010). It markedly decreases the survival rates of both the primary cancer cell population and the subpopulations of cancer stem cells (Czyz et al., 2013; Carlisi et al., 2016). The anticancer effects of PTL are not limited to increasing cell death; they also include anti-angiogenesis and anti-metastasis activities (Shields, 2017). The mechanism of PTL’s anticancer activity is complex. Indeed, PTL has multiple anti-leukemic mechanisms, including the activation of P53, autophagy, ROS (Reactive Oxygen Species), and the inhibition of STAT signal transducer and activator of transcription and NF-κB (Nuclear Factor-Kappa B) (Sztiller-Sikorska and Czyz, 2020; Kwok et al., 2001b). NF-κB is critically involves in various types of cancer and regulates the expression of genes essential for tumor growth, angiogenesis, and metastasis (Nagel et al., 2014; Thu et al., 2012). Elevated NF-KB (Nuclear Factor Kappa b) activity can significantly influence the responsiveness of cancer cells to therapy and play a role in drug resistance among malignant cells (Tian et al., 2019). Thus, this study aimed to explore the emerging function of Parthenolide extract as a modulator of signaling pathways in different hematological malignancies.

## 2 Search methodology

A systematic literature search across multiple databases, including PubMed, Scopus, Embase and Web of Science was done to identify relevant studies on PTL and its effects on hematological malignancies. The search strategy was composed of a combination of keywords and MeSH terms, such as “Parthenolide,” “hematological malignancies,” “AML,” “ALL,” “CML,” “CLL,” “MM,” “Lymphoma” and also each signaling pathway such as “apoptosis,” “autophagy,” and “NF-B”. Clear inclusion criteria were established, focusing on peer-reviewed studies that examined PTL’s mechanisms of action and therapeutic potential, while non-hematological studies and those lacking methodological rigor were excluded. Studies were prioritized based on their relevance and quality, ensuring a comprehensive overview of PTL’s role in treating hematological malignancies.

## 3 Multi-target antitumor mechanisms of parthenolide

PTL has emerged as a promising therapeutic agent due to its multi-targeted mechanisms of action that affect key signaling pathways involved in tumor survival and proliferation.

• Inhibition of NF-κB

PTL effectively inhibits the NF-κB signaling pathway, a crucial regulator of cell survival and inflammation. It achieves this by directly interacting with IKK (IκB-kinase), leading to reduced phosphorylation of IκB-alpha and decreased nuclear translocation of the p65 subunit of NF-κB (Guzman et al., 2005). This inhibition not only triggers apoptosis but also decreases the expression of ICAM-1, a downstream target of NF-κB, which may affect cancerous cell interactions with their environment (Minhajuddin et al., 2011). Moreover, PTL has been shown to disrupt the phosphorylation of signal transducers and activators of STAT3 at Tyr705, thereby reducing its activity and contributing to its antitumor effects (Garcia-Pineres et al., 2004; Sobota et al., 2000; García-Piñeres et al., 2001; García-Piñeres et al., 2004).

• Activation of p53

PTL also plays a significant role in the activation of the tumor suppressor protein p53. Studies indicate that exposure to PTL results in the phosphorylation of p53 at serine-15 (Guzman et al., 2005). The activation of p53 leads to increased expression of pro-apoptotic factors and contributes to the induction of apoptosis in malignant cells (Guzman et al., 2005).

• Increase in Reactive Oxygen Species

Another critical mechanism through which PTL exerts its antitumor effects is the induction of oxidative stress via increased levels of ROS. PTL enhances ROS production, which is essential for triggering apoptosis in cancer cells (Ren et al., 2022). Elevated ROS levels can lead to mitochondrial dysfunction, activating apoptotic pathways and causing cell death in various leukemia cell lines, including K562 and Kcl-22 (Ren et al., 2022). Additionally, PTL’s ability to induce ROS is linked to the modulation of the glutathione system, which further supports its cytotoxic effects (Guzman et al., 2005).

• Epigenetic Modulation

PTL has also been identified as a novel epigenetic modulator that inhibits DNA methyltransferase 1 (DNMT1) activity, leading to increased histone acetylation and upregulation of p21 (Flores‐Lopez et al., 2018). This mechanism suggests that PTL can alter gene expression profiles in cancer cells, enhancing their sensitivity to other therapeutic agents.

## 4 Parthenolide as a selective agent against drug-resistant stem cells

Recent research indicates that leukemia stem cells (LSCs), typically in a quiescent state, show little response to standard chemotherapy, which mainly targets actively dividing cells. Furthermore, existing treatments often fail to differentiate between normal and cancerous cells. This highlights the need for less toxic, more targeted therapies to address the drug-resistant characteristics of LSCs while preserving normal hematopoiesis (Pei et al., 2016).

Studies have demonstrated that PTL triggered substantial apoptosis in primary human acute myeloid leukemia (AML) cells and blast crisis chronic myeloid leukemia (bcCML) cells, while sparing normal hematopoietic cells (Guzman et al., 2005). In 2007, Guzman et al. showed that PTL and DMAPT (Dimethylamino Parthenolide) could effectively eliminate primary leukemia cells while sparing normal stem and progenitor cells. These findings suggest that PTL and similar sesquiterpene lactones may represent a new class of agents for targeting myeloid LSCs (Guzman et al., 2007a). Many studies suggest that PTL inhibits NF-κB by targeting IKK, affecting p50 and p65 subunits, disrupts STAT3 by blocking Tyr705 phosphorylation, enhances JNK (c-Jun N-terminal Kinase) activation, and increases intracellular ROS (Garcia-Pineres et al., 2004; Sobota et al., 2000; García-Piñeres et al., 2001; García-Piñeres et al., 2004). One study found that exposure to PTL results in the phosphorylation of p53 at serine-15, signifying its activation in AML stem cells. Notably, PTL also decreases the expression of ICAM-1 (Intercellular Adhesion Molecule 1) (Minhajuddin et al., 2011). A modified PTL analog, specifically altered at the C1–C10 positions, was found to effectively inhibit AML-LSCs. This compound also enhanced ROS production while demonstrating reduced toxicity to healthy bone marrow cells (Kempema et al., 2015). In a study conducted by Juan et al., PTL effectively eliminated drug-resistant leukemia stem cells and enhanced the sensitivity of K562/ADM cells to doxorubicin-induced apoptosis by down-regulating P-glycoprotein expression (Yi et al., 2021).

### 4.1 The effect of PTL on chronic myeloid leukemia cells

Chronic myeloid leukemia (CML) is a type of blood cancer characterized by a specific chromosomal alteration referred to as the Philadelphia chromosome (Ph). This change results from a translocation between chromosomes 9 and 22, creating the BCR-ABL (Breakpoint Cluster Region (BCR), Abelson murine Leukemia (ABL)) oncogene, which produces an active tyrosine kinase oncoprotein. This unique aspect of CML facilitates the use of targeted treatments known as tyrosine kinase inhibitors (TKIs) (Jabbour and Kantarjian, 2018).

PTLA-1, a Parthenolide analog, exhibited significant inhibitory effects on K562/ADR cells, leading to G0/G1 phase arrest by downregulating the expressions of Cyclin B1/D1 and CDK1/2/4/6 (Cyclin-dependent kinases). It initiated apoptosis through the mitochondrial pathway, impacting mitochondrial membrane potential (ΔΨm) and modulating proteins like Bcl-2, Bax (BCL2 Associated X), cytochrome c, and caspases 9 and 3 (40). PTL and DMAPT effectively induced apoptosis in CML cells (K562 and Kcl-22) by inhibiting p65 phosphorylation and increasing ROS levels (Flores-Lopez et al., 2018). Guzman et al. suggested that the effects of PTL and DMAPT on CML cells, encompassing both primitive primary cells and cell lines, can be linked to two primary pathways. These pathways include the inhibition of NF-κB and the production of ROS through modulation of the glutathione system (Guzman et al., 2005). Flores-Lopez et al. (2018) reported that PTL and DMAPT successfully targeted bulk CML cells and the CD34^+^ lin-population by increasing ROS levels and inhibiting the NF-κB pathway. Furthermore, PTL boosted the expression of Heme Oxygenase-1 (HMOX-1) through the erythroid nuclear factor 2 (Nrf2) as transcription factor, which allowed some CML cells to evade death partially. This led to cell cycle arrest in the G2/M and G0 phases, accompanied by changes in Cyclin D and A levels, as well as a reduction in CDK2 levels. Overall, PTL and DMAPT induce apoptosis in CML cells (Flores-López et al., 2016; Flores‐Lopez et al., 2018). PTL was identified as a novel epigenetic modulator in leukemia cell lines MV4-11, Kasumi-1, and K562. PTL inhibited DNMT1 activity and protein levels while down-regulating DNMT1 transcription. The mechanism involved covalent binding to DNMT1’s active site, leading to increased histone acetylation, potentially through HDAC (histone deacetylase) inhibition, which correlated with upregulation of p21. These findings suggest that PTL could be a promising agent for further studies in combination with other DNMT and HDAC inhibitors (Dai et al., 2010). A study investigated the role of myeloperoxidase (MPO) in PTL-induced cell death. K562/MPO cells showed significantly higher rates of apoptosis and mitochondrial membrane potential disruption than parental K562 cells following PTL treatment. PTL also increased ROS levels and downregulated anti-apoptotic proteins in K562/MPO cells, resulting in caspase activation (Kim SJ. et al., 2008).

### 4.2 Parthenolide in acute myeloid leukemia: mechanisms of action and therapeutic efficacy

AML is an aggressive cancer of myeloid white blood cells, driven by mutations in myeloblasts that disrupt differentiation and promote uncontrolled growth (Belloucif and Lobry, 2022). Key initiators of AML are leukemic stem cells, identified as CD34^+^ and either CD38^−^or CD38^+^. Despite advances in chemotherapy, AML has high relapse rates and poor survival outcomes. Current treatments primarily target cell cycle molecules but often result in significant side effects. Therefore, alternative therapies using herbal extracts may improve treatment efficacy while reducing adverse effects (Döhner et al., 2015; De Kouchkovsky and Abdul-Hay, 2016).

In a 2019 study by Noureldien et al., researchers developed poly (lactide-co-glycolide) (PLGA) nanoparticles linked with anti-CD44 to encapsulate PTL as an NF-kB inhibitor. This formulation specifically targeted leukemic cells while sparing normal cells. *In vitro* assays on Kasumi-1, KG-1a, and THP-1 cell lines revealed a 40% reduction in cell proliferation. In summary, PLGA-antiCD44-PTL nanoparticles significantly enhanced drug absorption and specifically targeted leukemic cells (Darwish et al., 2019). Combining PTL with agents that inhibit the Nrf2 response (2-deoxyglucose) and NADPH production (temsirolimus) creates a triple drug regimen (PDT) of PTL. This regimen shows significant toxicity against both bulk and stem/progenitor AML cells while sparing normal cells, even in resistant cases (Pei et al., 2016). Furthermore, several studies have indicated that inhibitors of the phosphoinositide 3-kinase (PI3K)/mTOR pathway, when used alongside PTL, significantly contribute to the selective destruction of primary human AML cells. The combination of PTL with the PI3K inhibitor wortmannin or the mTOR inhibitor rapamycin showed significant toxicity against AML stem cells, both *in vitro* using patient samples and in mouse models (Pei et al., 2016; Hassane et al., 2010). Additionally, since abnormal activation of FMS-like tyrosine kinase 3 (FLT3) is common in AML patients, the selective FLT3 inhibitor SC-203048 can work synergistically with PTL to suppress AML growth and enhance cellular apoptosis (Wang et al., 2012). A total of 34 new PTL derivatives were developed and tested for their anti-AML properties. Among these, 15 compounds demonstrated higher potency than PTL against the HL-60 cell line, while 21 outperformed PTL against the chemotherapy-resistant KG1a cell line. The study identified that various modifications at the C-14 position support strong anti-AML activity in the form of apoptosis induction (Yang et al., 2017). A series of PTL-SAHA hybrids was assessed for their anti-AML effects on HL-60 and HL-60/ADR cell lines. One particular hybrid induced apoptosis via the mitochondrial pathway, resulting in a dose-dependent decrease in HDAC1 and HDAC6 levels, as well as a reduction in ABCC1 (ATP Binding Cassette Subfamily C Member 1) expression. This mechanism enhanced the accumulation of drugs within cells and aided in overcoming resistance (Ge et al., 2019). PTL clinical use is hindered by rapid blood clearance due to hydrophobicity. Micelles (PSMA100-b-PS258) were created to enhance solubility, achieving a loading efficiency of 75% and enabling sustained release over 24 h. *In vitro*, PTL-loaded micelles were taken up by MV4-11 AML cells, displaying dose-dependent cytotoxicity that reduced cell viability by 75% and also inhibited NF-KB at 10 μM (Baranello et al., 2015). PTL effectively induced apoptosis while reducing OPN (osteopontin) expression in U937 cells, suggesting its potential as a therapeutic agent in AML (55). PTL notably enhanced the cytotoxic and pro-apoptotic effects of Ara-C (Cytosine arabinoside) in the HL-60 cell line (Papież and Krzyściak, 2018). A research was conducted to create dithiocarbamate esters of PTL to evaluate their anti-acute myelogenous leukemia (AML) properties. One particular ester demonstrated significantly greater potency against the KG1a AML progenitor cell line, inducing apoptosis in primary human AML cells and leukemia stem cells while leaving normal cells unharmed and effectively inhibiting the colony formation of leukemia cells (Ding et al., 2018). Wang et al. conducted research examining the impact of PTL on apoptosis induced by aclarubicin in human HL-60 leukemia cells. Their findings indicated that combining low doses of aclarubicin with PTL notably increased apoptosis rates. Additionally, this combination demonstrated a synergistic effect in inhibiting the activities of caspase 3, caspase 9, Cox-2 (Cyclooxygenase-2), and NF-κB (Wang et al., 2011). A study conducted in 2013 revealed that vildagliptin, an agent used to lower blood sugar levels, significantly increased the cytotoxic effects of PTL on the TEX leukemia stem cell line. This combination led to decreased cell viability and reduced clonogenic growth in cells from patients with acute myeloid leukemia while leaving normal peripheral blood stem cells unharmed. The observed synergistic effect was associated with the inhibition of DPP8 and DPP9(18). PTL could enhance the cytotoxic effects of etoposide by increasing GSH (glutathione) production in HL-60 cells (Papież et al., 2020). A study showed that KG1a cells were resistant to PTL, but suppressing Osteopontin could enhance PTL’s cytotoxicity. Combined treatment decreased AKT, mTOR, β-catenin, and PTEN (Phosphatase and tensin homolog) expression (Mohammadi et al., 2017). PTL also lowered the levels of eIF4E binding protein 1 (4E-BP1), which correlated with the induction of autophagy in the HL-60 cell line. Manipulating 4E-BP1 confirmed its role in this process. Additionally, PTL increased ROS, which preceded the reduction of 4E-BP1 and contributed to autophagy (Lan et al., 2015).

Ciclopirox, an antifungal agent, significantly enhances the efficacy of the antileukemia compound PTL against AML in the Kasumi-1 cell line by inhibiting mTOR, showing greater toxicity in combination than either agent alone (Sen et al., 2013). Shuangshuang et al. examined how PTL functions in human leukemia monocytic THP-1 cells and primary monocytes induced by LPS (lipopolysaccharide). They found that LPS significantly elevated levels of interleukin (IL)-6, IL-1β, IL-8, IL-12p40, tumor necrosis factor-α, IL-18, and nitric oxide, but PTL was able to inhibit these increases in a dose-dependent manner. Furthermore, PTL reduced the expression of TLR4 at both the protein and mRNA levels. It also inhibited the phosphorylation of several essential signaling proteins, including extracellular signal-regulated kinase 1/2, Jun N-terminal kinase, p38, NF-κB p65, and IκBα. Additionally, PTL decreased the expression of inducible nitric oxide synthase, TLR4, and TNF receptor-associated factor 6 in a dose-dependent manner (Li et al., 2015). PTL was found to enhance ATRA-induced differentiation of HL-60 cells into granulocytic cells. The signaling kinases PKC (Protein Kinase C), ERK (Extracellular signal-regulated kinase), JNK and PI3 were involved in the differentiation process enhanced by PTL. This enhancement of differentiation was associated with reduced NF-κB DNA-binding activity and occurred without raising basal intracellular calcium levels induced by ATRA (All-Trans Retinoic Acid) (Kim S. et al., 2008). Parthenolide inhibits nuclear factor-κB activation and shows anti-tumor effects in K562, Kasumi-1, and MV4-11 leukemia cell lines. PTL also reactivates the tumor suppressor gene HIN-1 (High in normal-1) via promoter hypomethylation (Liu et al., 2009). PTL has been reported to deplete glutathione and induce cell death in CD34^+^ AML cells, with minimal effects on normal cells (Pei et al., 2013). Therefore, Parthenolide, in combination with other drugs, can be beneficial in the treatment of AML patients.

## 5 Lymphoid malignancies: disruption in differentiation and the potential of parthenolide

Alterations in blood cell differentiation, resulting from obstructions, resistance to apoptosis, or heightened proliferation, can contribute to the development of stage-specific neoplasms. Lymphoid malignancies encompass a range of hematological neoplasms originating from the neoplastic transformation of B, T, and NK cells at various stages of differentiation (Kwok et al., 2001b; Wang et al., 2011). The development of lymphoid cells is contingent upon the differentiation of specific primitive cells within the bone marrow. In this setting, the early lymphoid progenitor (ELP) population plays a vital role in generating essential components of the innate immune system, such as plasmacytoid dendritic cells (pDC) and interferon-producing killer dendritic cells (IKDC). As these progenitors mature, they transform into common lymphoid progenitors (CLP), giving rise to precursors for B lymphocytes and NK cells, while losing the ability to differentiate into other cell types (Papież et al., 2020).

Despite advances in understanding lymphoid malignancies and developing targeted treatments, conventional chemotherapy remains inadequate, often resulting in chemoresistance, long-term toxicity, and relapse. This highlights the need for new therapeutic options. Parthenolide is a promising candidate due to its dual anti-tumor effects. It selectively targets the NF-κB signaling pathway and induces oxidative stress to eliminate malignant cells, potentially enhancing anti-tumor activity against progenitor lymphoid cells (Belloucif and Lobry, 2022; Mohammadi et al., 2017).

PTL showed considerable toxicity at a concentration of 10 μM in LSCs associated with acute lymphoblastic leukemia (ALL). In most cases, except for those with +3 karyotype abnormalities and CD34-CD38-CD19- LSCs with t (Cheon, 2021; Carlisi et al., 2016), over 50% cell death was observed at a concentration of 25 μM. Notably, LSCs that were CD34^+^, CD38^+^, and CD19^+^ showed greater sensitivity to PTL (Lan et al., 2015).

### 5.1 PTL induces selective apoptosis of chronic lymphocytic leukemia (CLL)

In chronic lymphocytic leukemia (CLL), malignant CD5^+^ CD19^+^ cell clones accumulate by avoiding programmed cell death. CLL is mainly categorized into two subtypes that present notably different clinical outcomes. About two-thirds of cases belong to a favorable prognostic group, characterized by hypermutation in the immunoglobulin heavy chain variable (IgVH) gene segments. On the other hand, the remaining one-third of patients possess relatively unmutated IgVH genes, which are associated with a worse prognosis, leading to swift disease progression and often fatal results (Liu et al., 2009; Pei et al., 2013).

Chlorambucil has been the standard treatment for CLL for many years. Nevertheless, using this drug on its own does not markedly improve patients’ overall survival rates. Additionally, it can be highly detrimental to T lymphocytes, increasing the risk of opportunistic infections due to the prolonged suppression of CD4^+^ T cell levels (Liu et al., 2009). The progressive emergence of drug resistance following treatment with DNA-damaging agents restricts their effectiveness in therapy (Wickremasinghe et al., 2011). Marin and Mansilla (2006) studied the effects of PTL on inducing apoptosis and cytotoxicity in B-CLL cells *in vitro*. The study found that PTL effectively triggers apoptosis and exhibits potent cytotoxic effects specifically on B-CLL cells. Notably, it had little to no effect on normal peripheral blood mononuclear cells (PBMCs). This suggests that PTL may represent a promising therapeutic strategy for B-CLL (Marin and Mansilla, 2006).

PTL exhibits effective and selective anti-CLL activity *in vitro*, targeting CLL cells irrespective of their p53 status, owing to its pro-oxidant properties (Agathanggelou et al., 2015). Studies indicate that CLL cells are more responsive to PTL than normal T lymphocytes or CD34^+^ hematopoietic progenitor cells. The mechanism involves PTL-induced production of reactive oxygen species, which triggers changes in the pro-apoptotic protein Bax, leading to the release of cytochrome c from the mitochondria and the activation of caspases (Steele et al., 2006).

Recent studies show that redox-activated small molecules selectively have a cytotoxic effect on CLL. The factor 2 signaling pathway associated with Nrf2 regulates the oxidative stress response (Wu et al., 2010; Rushworth and MacEwan, 2011). Wu et al. (2010) examined Nrf2 signaling in untreated CLL cells in comparison to normal lymphocytes, revealing that compounds with α-β unsaturated carbonyls, sulfhydryl-reacting metals, and isothiocyanates serve as potent Nrf2 activators. Elevating Nrf2 expression, associated with heme oxygenase-1, could enhance the cytotoxic effects against CLL. Substances like atacrinic acid and PTLwere shown to activate Nrf2 in peripheral blood mononuclear cells, indicating that changes in Nrf2 responses may contribute to the selective cytotoxicity seen in CLL (Wu et al., 2010).

### 5.2 Parthenolide-induced apoptosis in acute lymphoblastic leukemia: mechanisms and therapeutic potential

Acute lymphocytic leukemia is a cancer characterized by the uncontrolled growth of immature B or T lymphoblasts. This proliferation replaces normal bone marrow and impacts other lymphatic organs, resulting in a distinct disease pattern. In the United States, ALL accounts for approximately 2% of lymphoid neoplasms (Marinescu et al., 2015).

In T-cell acute lymphoblastic leukemia (T-ALL), elevated ROS inhibit PTEN, enhancing leukemia cell survival. ROS levels are regulated by decreased PKCθ due to NOTCH-1 (Neurogenic locus notch homolog protein 1) mutations, and excessive ROS can trigger cell death. This suggests that ROS boosters may effectively target cancer cells, although the exact mechanism of PTL toxicity in T-ALL is not fully understood (Diamanti et al., 2013; Ede et al., 2018). PTL was found to effectively trigger apoptosis in bulk B- and T-ALL cells in the xenograft model, though some leukemia-initiating cells subpopulations (CD34^+^/CD19^−^, CD34^+^/CD7^−^, and CD34^−^) showed greater resistance (Diamanti et al., 2013).

Zunino et al. (2010) examined cell cycle status and phosphorylation/activation of signaling proteins in acute lymphoblastic leukemia cell lines after treatment with PTL. The cells were treated with 10 μM PTL for 2, 4, 6 and 8 h. PTL stopped growth in S to G2/M phase transition (Zunino et al., 2010).

PTL demonstrates cytotoxic and apoptotic activity against Jurkat cells as T-cell ALL, with an IC50 of 16.1 μM (Mehri et al., 2020). In a 2007 study by Zunino, PTL was shown to induce rapid apoptosis in pre-B ALL cell lines, including those with the t (Rusu et al., 2018; Lin et al., 2020) (q21; q23) translocation. The apoptotic effects were characterized by nuclear DNA fragmentation, externalization of phosphatidylserine, increased levels of nitric oxide and superoxide anions, and mitochondrial membrane depolarization at concentrations ranging from 5 to 100 μM (Zunino et al., 2007). Jorge et al. investigated the impact of PTL on leukemic cell lines, including KOPN-8 (B-ALL), CEM, and MOLT-4 (T-ALL). They found that PTL induces apoptosis through caspase activation, causes cell cycle arrest at the G2/M phase, increases ROS production, lowers GSH levels, activates caspase-3 and FAS-ligand, and reduces pNF-κB expression (Jorge et al., 2023). PTL notably enhanced the toxicity of arsenic trioxide (ATO) in treating MT2 cell line as T-ALL. Furthermore, molecular analysis indicated significant downregulation of CD44, NF-κB (REL-A), BMI-1, and C-MYC when PTL was combined with ATO (Kouhpaikar et al., 2020). A study of 11 cases of B cell precursor (BCP) acute lymphoblastic leukemia found that the combination of PTL and the BCL-2 inhibitor ABT-263 significantly reduced cell viability. *In vivo* experiments with NSG mice demonstrated that ABT-263 effectively decreased leukemia burden and improved survival compared to the control group (Diamanti et al., 2015). Research indicated that PTL effectively targets both CD200+ and CD200− subpopulations in low-risk and high-risk NSG mice models of BCP-ALL. Although the presence of mesenchymal stem cells (MSCs) diminished sensitivity to PTL, this effect was significantly restored with sulfasalazine (SSZ), a cystine uptake transporter inhibitor. This restoration was particularly notable in CD34+/CD200+ cells in low-risk cases and CD34−/CD200+ cells in high-risk cases (Diamanti et al., 2018).

A 2024 study demonstrated the effects of PTL, DMAPT, and PU-H71 (Purine-scaffold inhibitor) on pediatric B-ALL using untreated bone marrow samples. PTL and DMAPT significantly reduced B-ALL cells while having minimal effects on normal cells, whereas PU-H71 also decreased the leukemic population but had a lesser sparing effect on normal cells. These results indicate that PTL and DMAPT may serve as promising treatments for B-ALL, potentially preventing disease progression or relapse (Ortiz-Reyes et al., 2024).

These data suggest that PTL elicits a stress response that leads to cell death and provides further evidence that PTL can be used as a new therapeutic agent against acute lymphoblastic leukemia.

## 6 Parthenolide’s mechanism of action against multiple myeloma cells

Multiple myeloma (MM), commonly referred to as Kahler’s disease, is a plasma cell malignancy that remains an incurable form of chronic leukemia, despite the availability of various treatment options. The interactions between MM cells and the bone marrow microenvironment influence the varying responses to therapy and contribute to the development of drug resistance. The nuclear factor-κB (NF-κB) family, particularly the p65/p50 heteromer, is essential in the pathogenesis of MM. When MM cells bind to bone marrow stromal cells (BMSCs), the activation of NF-κB influences the production of IL-6 and vascular endothelial growth factors, thereby facilitating the proliferation of MM cells (Mitsiades et al., 2006; Karin, 2006; Rajkumar and Kumar, 2020).

A study indicated that both Parthenolide and andrographolide exhibited greater toxicity toward MM-CSCs compared to non-tumorigenic MM cells in both 2D and 3D cultures (Gunn et al., 2011). Wang et al. (2006) discovered that PTL effectively triggers apoptosis in MM cell lines and primary MM cells that express high levels of CD38^+^. This apoptotic process is strongly associated with generating ROS and can be almost entirely blocked by L-N-acetylcysteine (L-NAC). Additionally, decreasing catalase activity using siRNA heightened the sensitivity of the cells to PTL. These findings indicate that the apoptosis induced by PTL in MM cells is dependent on increased ROS levels, with intracellular catalase activity playing a key role in determining their sensitivity to the treatment (Wang et al., 2006). Kong et al. (2015) explored how PTL inhibits NF-κB activity in MM. Their findings revealed that treating myeloma cells with PTL led to a reduction in ubiquitin levels, an increase in IκB-α expression, and a decrease in nuclear p65 levels, ultimately resulting in diminished NF-κB activity. PTL was found to inhibit cell proliferation, induce apoptosis, and disrupt the cell cycle. Furthermore, after PTL treatment, levels of ubiquitinated tumor necrosis factor receptor-associated factor 6 (TRAF6) and total protein were found to decrease (Kong et al., 2015). In a study working on RPMI8226 cells as a MM cell line,PTL decreases NF-kB activity and significantly lowers the expression of VEGF (Vascular endothelial growth factor) and IL-6 mRNA and protein, which may contribute to its anti-angiogenic effects (Kong et al., 2008). Suvannasankha et al. conducted a study about the effect of PTL on H929, ARH-77, IM-9, U266 and RPMI-8226 cell lines as multiple myeloma cells. PTL inhibited interleukin-6 secretion from bone marrow stromal cells activated by MM cell adhesion. This extract activated caspases and cleft key proteins like PARP (poly-ADP ribose polymerase) and MCL-1 (myeloid cell leukemia 1). Also combined treatment of PTL with dexamethasone resulted in additive and synergistic effects (Suvannasankha et al., 2008). A 2008 study evaluated the effects of LC-1 (a PTL derivative) on multiple myeloma cell lines, including H929, U266, and JJN3. LC-1 showed cytotoxicity with LD50 values of 3.6 mM for MM cell lines. LC-1 reduced NF-κB nuclear localization and RelA DNA binding. Synergy was reported when combined with melphalan and bortezomib, but not with doxorubicin (Walsby et al., 2008).

In conclusion, PTL induces apoptosis in multiple myeloma cells by increasing ROS and inhibiting NF-κB activity. It reduces NF-κB nuclear localization and angiogenic factor expression, highlighting its therapeutic potential.

## 7 Pharmacokinetic and pharmacodynamic challenges of PTL

Although no clinical trials have been conducted to date on the effects of PTL in patients with hematological malignancies, preclinical studies have shown that PTL effectively inhibits NF-kB and selectively targets leukemic cells. However, its poor water solubility limits bioavailability and complicates dosing. To address this challenge, researchers have developed analogs such as DMAPT, which is approximately 1000-fold more soluble than PTL while retaining its activity against leukemia stem cells (Jordan, 2007; Li et al., 2019). Research using mouse xenograft models and spontaneous acute leukemia in dogs indicated that DMAPT possesses *in vivo* bioactivity, demonstrated through functional assays and various biomarkers. Although DMAPT moved forward to phase I clinical trials for AML treatment in the United Kingdom, its low water solubility and metabolic instability ultimately caused the trial to fail (Guzman et al., 2007b). Some researchers have also used liposomes to overcome the solubility challenges associated with PTL (Nguyen et al., 2010).

Due to the low stability of PTL, some researchers have utilized various vectors, nanoparticles, and micelles to address this issue (Zong et al., 2016). In a 2018 study, researchers had used safe nano-vectors with direct hydration method to treat T-ALL and B-ALL cells with PTL stabilized. The PTL nanovectors showed remarkable stability over 3 weeks in physiological conditions and released 80%–90% of the drug within 5 h without a burst effect. *In vitro* assays revealed significant cytotoxicity against various ALL patient samples while sparing healthy cells, confirming their biocompatibility (Ridolfo et al., 2018). PTL poses rapid blood clearance due to hydrophobicity, but micelles made from poly (styrene-alt-maleic anhydride)-b-poly (styrene) enhance its solubility, achieving 75% loading efficiency and sustained release over 24 h. *In vitro* studies demonstrated effective uptake by MV4-11 AML cells, resulting in a 75% reduction in cell viability at 10 μM PTL, with micelle-mediated delivery maintaining cytotoxic effects over 24 h compared to free PTL (Baranello et al., 2015). In a 2019 study, researchers employed targeted nano-encapsulation with PLGA nanoparticles conjugated to anti-CD44 to enhance drug delivery and selectively target leukemic cells. This method significantly increased the bioavailability of PTL, achieving a 40% reduction in leukemic cell proliferation compared to free PTL (Darwish et al., 2019).

It is important to note that some studies have utilized hybrid or pro-drug forms of PTL while some researchers prefer to use combination treatment. In a study conducted by Taleghani et al., parthabine (a hybrid of parthenolide and cytarabine) and parthalan (a hybrid of parthenolide and melphalan) were synthesized, demonstrating enhanced cytotoxicity against various tumor cell lines with lower IC50 values compared to their parent compounds (Taleghani et al., 2017). Table 1 summarizes the preclinical studies involving PTL, highlighting the effective concentrations used in combination with other drugs or in different formats.

**TABLE 1:** Studies investigating the effect of parthenolide on hematological malignancies.

Hematological malignancy	Cell line/Animal model	Mechanism of PTL	Effective concentration of PTL	Selected drug for combination treatment	Year	Ref
AML	AML cells/(NOD/SCID) mouse	Reduction of NF-KB Increase of P53 and ROS	7.5 µM after 18 h	PGJ2 (15-deoxy-delta12,14-prostaglandin J2)	2005	Guzman et al. (2005)
AML and ALL	Primary human AML, bcCML,ALL cells and U937 cell line/(NOD/SCID) mouse	Reduction of NF-KB Increase of P53 and ROS	7.5 µM after 18 h	—	2007	Guzman et al. (2007a)
ALL-T	Jurkat T-cells and HeLa cells	Reduction of NF-KB Alkylating P65	25 µM after 1 h	Cycloheximide,N-ethyl-maleimide (NEM) and TNF-α	2004	Garcia-Pineres et al. (2004)
ALL-T	Jurkat T-cell and 293 cells	Reduction of NF-KB and P65	5–20 µM after 1 h	4 B,15-epoxy-miller-9E-enolide	2001	García-Piñeres et al. (2001)
APL and ALL-T	HL-60 and CCRF-CEM	Increase of ROS	25 µM after 12 h	—	2015	Kempema et al. (2015)
AML and CML	Meg‐01, K562, Kasumi‐4, KCL‐22 cells and HL-60	Reduction of NF-KB Increase of ROS Cell cycle arrest G0/G2	5 and 7.5 µM after24-48 h	—	2018	Flores-Lopez et al. (2018)
AML	U937, HL-60, NB4, MV-4–11, MOLM-13 and MLL-ENL cells	Reduction of NF-KB Increase of SAPK/JNK	5–8 μM after 24 h	HDACIs (vorinostat)	2010	Dai et al. (2010)
CML	K562/MPO cells	Increase of ROS	10 μM after 24 h	—	2008	Kim et al. (2008a)
AML	Kasumi-1, KG-1a, and THP-1	Reduction of NF-KB	5 µM PLGA-antiCD44-PTL nanoparticles after 48 h	—	2019	Darwish et al. (2019)
AML	AML cells and NOD/SCID mouse	Increase of PI3K/mTOR	5 μM after 6 h	PI3K inhibitors	2010	Hassane et al. (2010)
AML	THP-1 cell line/BALB/c nude mouse	Reduction of FLT3, p65, cyclin D1 and Bcl-2 Increase of SMRT	10 μg/kg after 48 h	FLT3 inhibitors	2012	Wang et al. (2012)
AML	HL-60 and KG1a cell line	Increase of Apoptosis	10–20 µM after 16 h	—	2017	Yang et al. (2017)
AML	HL-60 and HL-60/ADR cell lines	Reduction of HDAC1, HDAC6, ABCC1 and BCl-2 Increase of Bax and Cyto C	0.5 µM after 24 h of PTL-SAHA hybrid	—	2019	Ge et al. (2019)
AML	MV4-11 AML cells	Reduction of NF-KB and GSH	10 µM after 24 h of Micelle encapsulated PTL	—	2015	Baranello et al. (2015)
AML	U937 cell line	Increase of Apoptosis Reduction of OPN	5.8 µM after 20–24 h	—	2016	Zahedpanah et al. (2016)
APL	HL-60 cell line	Increase of Apoptosis	5 µM after 24 h	Cytarabine	2018	Papież and Krzyściak (2018)
AML	HL-60 HL-60/adriamycin (ADR), THP-1, K562, and KG1a/Xenograft model of mice	Increase of Apoptosis and MAPK	0.7 µM after 72 h of dithiocarbamate esters of parthenolide	—	2018	Ding et al. (2018)
AML	HL-60 cell line	Decrease of GSH Increase of Apoptosis	5 µM after 24 h	Etoposide	2020	Papież et al. (2020)
AML	KG1a cell line	Increase of OPN Reduction of AKT, mTOR, β-catenin and PTEN	6 µM after 20–24 h	Daunorubicin	2017	Mohammadi et al. (2017)
AML	HL-60 cells, HeLa cells, and HEK293 T cells	Reduction of 4 E-BP1 Increase of ROS, Apoptosis and Autophagy	25 µM after 1.5 h	—	2015	Lan et al. (2015)
AML	Kasumi-1 cell line	Increase of mTOR Reduction of NF-KB	10 µM after 6 h	Ciclopirox Temsirolimus	2013	Sen et al. (2013)
AML	THP-1 cell line	Reduction of TLR-4, ERK1/2, JNK, p38, IL-6, IL-1β, IL-8, IL-12p40, TNF-α, IL-18 and NO	0.75–12 µM after 24 h	LPS	2015	Li et al. (2015)
AML	HL-60 cell line	Reduction of DNA synthesis, P85 and NF-KB Cell cycle arrest in G0/G1 Increase of PKC	10 µM after 12–72 h	ATRA	2008	Kim et al. (2008b)
AML	MV4-11 leukemia cell line	Reduction of DNMT1 Increase of HIN-1	3.5 µM after 24 h	—	2009	Liu et al. (2009)
AML	AML cells	Reduction of GCLC, GSH and GPX1	10 µM after 24 h	Cytarabine Idarubicine	2013	Pei et al. (2013)
CLL	B-CLL cells	Increase of Apoptosis	10 µM after 72 h	—	2006	Marin and Mansilla (2006)
CLL	CLL cells/NSG mouse	Increase of Apoptosis	6 mg/kg for 9 days	—	2015	Agathanggelou et al. (2015)
CLL	CLL cells	Increase of ROS, Bax, Cytochrom C, Caspase Reduction of NF-KB	6.2 µM after 6 h	—	2006	Steele et al. (2006)
CLL	CLL cells	Increase of NRf2	10 µM after 4 h	—	2010	Wu et al. (2010)
ALL	T-ALL cells	Reduction of GSH Increase of ROS	10 µM after 1 h	N-acetyl cysteine	2018	Ede et al. (2018)
ALL	SEM, RS4; 11 and REH cells	Cell cycle arrest S to G2/M Increase of p38 MAPK, c-Jun kinase, c-Jun,HSP27 and protein kinase B	10 µM after 8 h	—	2010	Zunino et al. (2010)
T-ALL	Jurkat cell line	Increase of Apoptosis	16.1 μM after 48 h	—	2020	Mehri et al. (2020)
B-ALL	SEM, RS4; 11 and REH cell lines	Increase of ROS and Apoptosis	100 μM after 24 h	—	2007	Zunino et al. (2007)
T-ALL B-ALL MM	NCI-H929 (MM) Farage (GCB-DLBCL) Raji (BL) KOPN-8 (B-ALL) CEM MOLT-4 (T-ALL)	Increase of ROS and Apoptosis Reduction of GSH and NF-KB	1 μM and 10 µM after 72 h	—	2023	Jorge et al. (2023)
ALL	MT2 cells ATLL cell line	Reduction of CD44, NF-κB (REL-A), BMI-1 and C-MYC	1 μg/mL after 72 h	ASO	2020	Kouhpaikar et al. (2020)
ALL	ALL cells/NOD/LtSz-scid IL-2Rγc null (NSG) mice	Reduction of NF-KB	1.2 μM after 24 h	BCL-2 inhibitors	2015	Diamanti et al. (2015)
ALL	BCP-ALL cells	Increase of Apoptosis and ROS	7.5 μM after 24 h	Sulfasalazine	2018	Diamanti et al. (2018)
ALL	RS4; 11 and Reh cell line	Increase of Apoptosis	5 μM after 24–48 h	DMAPT PU-H71	2024	Ortiz-Reyes et al. (2024)
MM	RPMI-8226 and U266 cell line	Increase of Apoptosis	20–100 μM after 48 h	—	2011	Gunn et al. (2011)
MM	MM1S, KMM-1, KMS-5 and NCI-H929 cell lines	Increase of Apoptosis and ROS	5 μM after 48 h	—	2006	Wang et al. (2006)
MM	RPMI 8226 cell line	Reduction of P65, NF-KB and TRAF-6 Increase of Apoptosis	40 μM after 24 h	—	2015	Kong et al. (2015)
MM	RPMI8226 cell line	Reduction of P65, VEGF, IL-6 and NF-KB Increase of IkappaB-alpha	3.5 μM–10 μM after 48 h	—	2008	Kong et al. (2008)
MM	MM.1 S MM1.R NCI-H929 ARH-77 IM-9 U266 RPMI8226 cell lines	Decrease of NF-KB, IL-6, *c-FLIP* and *TRAF2* Increase of Apoptosis	5 μM–10 μM after 48 h	—	2008	Suvannasankha et al. (2008)
MM	H929, U266 and JJN3 cell lines	Increase of Apoptosis Reduction of NF-KB	3.6 μM–5.4 μM after 48 h	Doxorubicin Melphalan Bortezomib	2008	Walsby et al. (2008)

Abbreviations: ABCC1 (ATP, Binding Cassette Subfamily C Member 1), AML (Acute Myeloid Leukemia), ALL (Acute Lymphoblastic Leukemia), APL (Acute Promyelocytic Leukemia), Bcl-2 (B-cell Lymphoma 2), Bax (BCL2 Associated X), bcCML (Blast Crisis Chronic Myeloid Leukemia), Bmi-1 (B cell-specific Moloney murine leukemia virus Integration site 1), COX-2 (Cyclooxygenase-2), c-FLIP (Cellular FLICE-Inhibitory Protein), DNMT1 (DNA, Methyltransferase 1), FLT3 (Fms-like Tyrosine Kinase 3), GCLC (Glutamate-Cysteine Ligase Catalytic Subunit), GPx-1 (Glutathione Peroxidase 1), GSH (Glutathione), HIN-1 (High in Normal 1), HDAC (Histone Deacetylases), HSP-27 (Heat Shock Protein 27), NOD/SCID (Non-Obese Diabetic/Severe Combined Immunodeficiency Mouse), NF-κB (Nuclear Factor Kappa B), OPN (Osteopontin), PI3K/mTOR (Phosphatidylinositol 3-Kinase/mammalian Target of Rapamycin), PKC (Protein Kinase C), PTEN (Phosphatase and Tensin Homolog), ROS (Reactive Oxygen Species), SMRT (Silencing Mediator of Retinoic Acid and Thyroid Hormone Receptor), TNF-α (Tumor Necrosis Factor Alpha), TRAF2 (Tumor Necrosis Factor (TNF) Receptor Associated Factor-2), VEGF (Vascular Endothelial Growth Factor), 4 E-BP1 (eIF4E Binding Protein 1).

## 8 Conclusion

**FIGURE 1:**
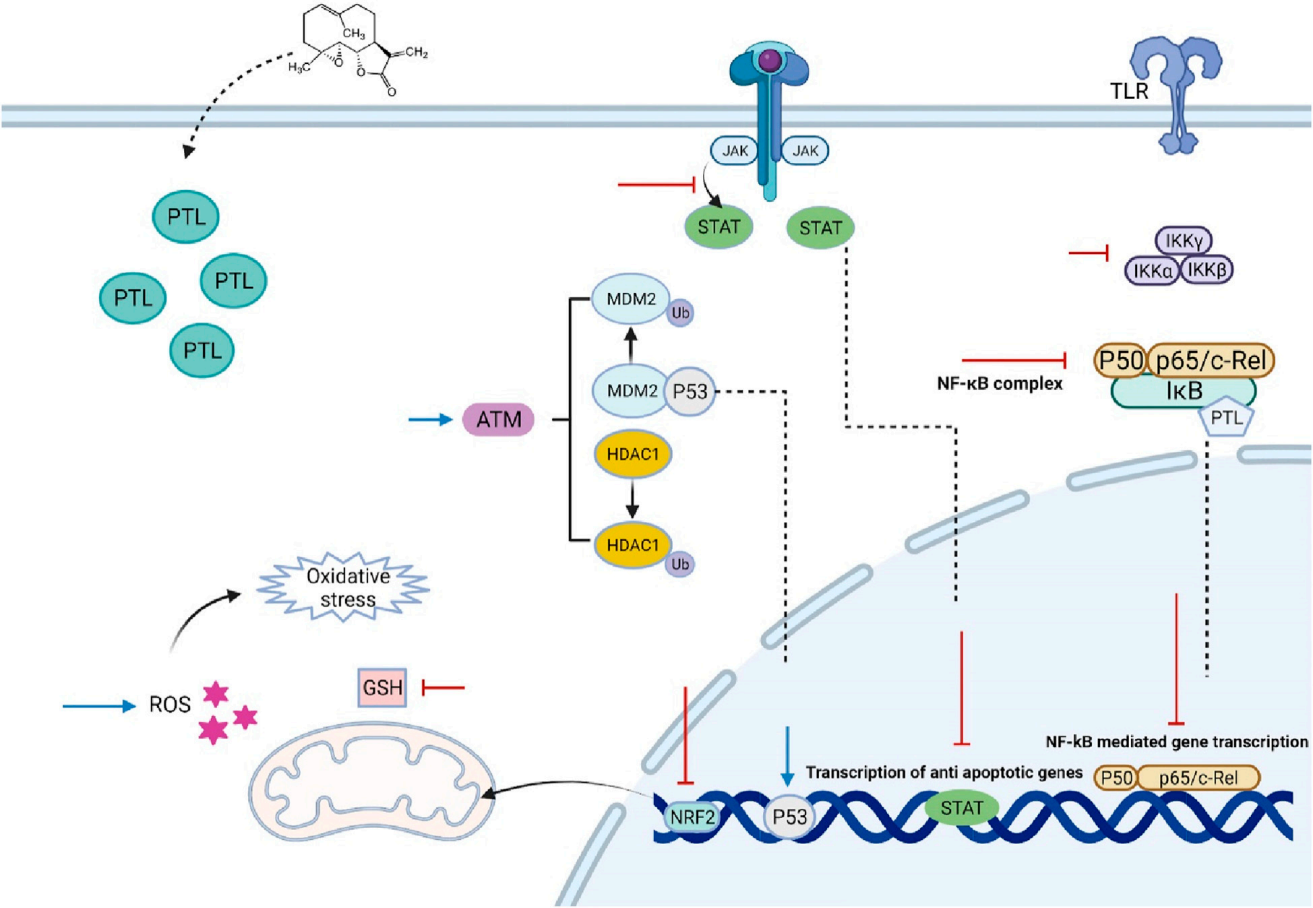
The effect of Parthenolide on various cancerous cells of hematological malignancies. PTL inhibits the upstream kinases of the IκB complex (IKK), preventing the degradation of NF-κB regulatory proteins IκB-α and IκB-β, and/or directly inhibiting the NF-κB/p65 subunit. It alkylates Cys38 and Cys120, leading to the inhibition of NF-κB activation, which is associated with sustained activation of C-Jun N-terminal kinase (JNK) and ultimately results in cancer cell death. PTL also alters p53 activity through two mechanisms involving the ataxia telangiectasia mutated serine/threonine kinase, either by inducing the degradation of MDM2, a negative regulator of p53, or by reducing histone deacetylase 1. Furthermore, PTL is a potent inhibitor of STAT proteins, blocking STAT3 phosphorylation at Tyr705, which prevents its dimerization, nuclear translocation, and gene expression. While PTL does not interfere with STAT6 phosphorylation, it inhibits its binding to DNA. Its therapeutic effects are linked to oxidative stress, inducing apoptosis by reducing intracellular thiol levels, including free glutathione (GSH), and producing reactive oxygen species (ROS). PTL also promotes ROS-mediated autophagy. Additionally, PTL acts as an effective DNA hypomethylating agent by inhibiting DNA methyltransferase 1. However, by activating the nuclear factor erythroid 2/antioxidant response elements pathway, PTL protects normal cells from oxidative stress while potentially aiding in the protection and growth of cancer cells. Abbreviations: ARE (Antioxidant Response Elements), ATM (Ataxia Telangiectasia Mutated), DNMT1 (DNA Methyltransferase 1), DNA (Deoxyribonucleic Acid), GSH (Glutathione), HDAC1 (Histone Deacetylase 1), IKK (IκB Kinase), IκB (Inhibitor of Kappa B), JNK (C-Jun N-terminal Kinase), MDM2 (Mouse Double Minute 2 Homolog), NF-κB (Nuclear Factor Kappa-light-chain-enhancer of activated B cells), Nrf2 (Nuclear Factor Erythroid Related Factor 2), p53 (Tumor Protein p53), PTL (Parthenolide), ROS (Reactive Oxygen Species), STAT (Signal Transducer and Activator of Transcription), Tyr705 (Tyrosine 705).

In conclusion, this review emphasizes the urgent need for innovative treatment strategies in managing hematological malignancies, which remain significant challenges owing to their complex biology and the limitations of current therapies. Traditional treatments, particularly chemotherapy, often result in severe side effects and inadequate responses, especially in the context of resistant leukemia stem cells and other malignant progenitor cells. The exploration of natural compounds, particularly Parthenolide, has emerged as a promising avenue in this landscape. PTL demonstrates a multifaceted mechanism of action that not only induces apoptosis in cancer cells but also modulates key signaling pathways involved in cell survival and resistance (Figure 1). Its ability to selectively target malignant cells while sparing normal hematopoietic cells positions PTL as a potential cornerstone in the development of safer and more effective therapeutic regimens. Moreover, the synergistic effects of PTL with conventional therapies underscore its potential to enhance treatment outcomes and overcome resistance mechanisms that complicate the management of hematological malignancies. Although PTL had been demonstrated as a good anti-leukemic agent but its clinical application is limited due to poor water solubility, which affects bioavailability and pharmacokinetics, complicating dosing regimens and potentially leading to suboptimal therapeutic outcomes. To address these limitations, researchers have found some strategies such as using analogs of PTL such as DMAPT, make liposome enveloped PTL, Ongoing research and clinical trials are vital to confirm PTL’s effectiveness as a treatment option. Ultimately, these innovative approaches have the potential to enhance survival rates and improve the quality of life for patients, marking a significant advancement in the fight against blood cancers.

## 9 Future prospective

As we deepen our understanding of hematological malignancies and their treatments, PTL, an herbal compound, emerges as a hopeful option. Chemotherapy drugs often come with significant side effects and face challenges like drug resistance, but PTL specifically targets leukemic stem cells. Insufficient pharmacokinetics and pharmacodynamics data for PTL limits its clinical use, with unclear information on absorption, distribution, metabolism, and excretion. There is also a lack of detailed studies on its dose-response relationships and therapeutic window. Additionally, toxicity data is scarce, making it difficult to assess potential side effects and long-term safety. It is crucial that we continue researching how PTL targets signaling pathways and how it can be effectively combined with other treatments. Upcoming clinical trials will be the key to determine how safe and effective PTL is for different patients. We hope that researchers will develop enhanced treatment strategies that not only increase survival rates but also improve the quality of life for individuals facing these challenging diseases.

## Glossary

ABCC1: ATP Binding Cassette Subfamily C Member 1
ALL: Acute Lymphoblastic Leukemia
AML: Acute Myeloblastic Leukemia
Ara-C: Cytosine arabinoside
ATO: Arsenic trioxide
ATRA: All-Trans Retinoic Acid
B-ALL: B Lymphocyte-ALL
B-CLL: B-cell Chronic Lymphocytic Leukemia
BAX: BCL2 Associated X
Bc-CML: Blast crisis-CML
BCR-ABL: Breakpoint Cluster Region (BCR), Abelson murine Leukemia (ABL)
BMSCS: Bone Marrow Stromal cells
CDK: Cyclin-dependent kinases
cFLIP: Cellular FLICE-like inhibitory protein
CLL: Chronic Lymphocytic Leukemia
CLP: Common Lymphoid Progenitors
CML: Chronic Myelocytic Leukemia
COX-2: Cyclooxygenase-2
DAMPT: Dimethylamino Parthenolide
DNMT-1: DNA (cytosine-5)-methyltransferase 1
DPP-8: Dipeptidyl peptidases 8
DPP-9: Dipeptidyl peptidases 9
eIF4E: Eukaryotic translation initiation factor 4 E
ELP: Early lymphoid progenitor
ERK: Extracellular signal-regulated kinase
FLT3: Fms-like tyrosine kinase 3
FAS ligand: FS-7-associated surface antigen ligand
GSH: Glutathione
HDAC: Histone deacetylases
HMOX-1: Heme Oxygenase-1
HIN-1: High in normal-1
HSP-27: Heat shock protein 27
IKK: IK-B Kinase
IKDC: Interferon-producing killer Dendritic cells
IC50: Half-maximal inhibitory concentration
ICAM-1: Intercellular Adhesion Molecule 1
IgvH: Immunoglobulin heavy chain variable
JNK: c-Jun N-terminal Kinase
L-NAC: L-N-acetyl cysteine
LD-50: median lethal dose
LPS: Lipopolysaccharide
LSC: Leukemic Stem Cells
MAPK: Mitogen-activated protein kinases
MM: Multiple Myeloma
MPO: Myeloperoxidase
Mcl-1: myeloid cell leukemia 1
Nfr2: Nuclear factor erythroid 2–related factor 2
NF-KB: Nuclear Factor Kappa B
NOTCH-1: Neurogenic locus notch homolog protein 1
OPN: Osteopontin
PARP: poly-ADP ribose polymerase
PBMC: Peripheral Blood Mononuclear Cell
Ph: Philadelphia chromosome
PI3K/mTOR: phosphatidylinositol-3-kinase/Akt and the mammalian target of rapamycin
PKC-Ɵ: Protein Kinase C Theta
PTEN: Phosphatase and tensin homolog
PTLA-1: Parthenolide analogue
PTL: Parthenolide
PU-H71: Purine-scaffold inhibitor
ROS: Reactive Oxygen Species
SMRT: Silencing mediator of retinoic acid and thyroid hormone receptor
siRNA: Small interfering RNAs
SSZ: Sulfasalazine
SP1: Specificity protein 1
STAT: Signal transducer and activator of transcription
TKI: Tyrosine Kinase Inhibitor
TLR-4: Toll-Like Receptor-4
TRAF: TNFR-associated factor 2
TRAF6: Tumor necrosis factor receptor associated factor 6
VEGF: Vascular endothelial growth factor
ΔΨm: Mitochondrial Membrane Potential
